# Response of vegetation phenology to hydrothermal variables on the QTP using EVI and MSAVI

**DOI:** 10.1016/j.isci.2026.115206

**Published:** 2026-03-03

**Authors:** Zhijian Zhao, Hui Lin, Li Wang, Min Huang, Lei Wu, Linling Tang, Tao Yang, Xin Xiao

**Affiliations:** 1 Key Laboratory of Poyang Lake Wetland and Watershed Research Ministry of Education/Postdoctoral Research Station of Chemistry, Jiangxi Normal University, Nanchang 330022, China; 2 College of Mathematics and Computer, Xinyu University, Xinyu 338004, China

**Keywords:** Botany, Earth sciences, Environmental science

## Abstract

Vegetation phenology is a key indicator of how the Qinghai-Tibet plateau (QTP) ecosystem responds to changes in the hydrothermal environment. However, sparse QTP meteorological stations make observed traditional hydrothermal variables (air temperature and precipitation) insufficient for research needs. Building upon analyses of vegetation phenology using enhanced vegetation index and modified soil-adjusted vegetation index, this study adopts alternative hydrothermal variables (land surface temperature [LST], effective moisture and surface albedo). Geodetector and partial correlation analysis were employed to reveal the response mechanisms between vegetation phenology and hydrothermal variables. Results indicate that the length of the growing season (LOS) is primarily driven by the end of the growing season (EOS). From 2001 to 2020, EOS showed a significant positive correlation with LST. Compared to traditional variables, these variables demonstrated stronger explanatory power for vegetation changes. Hurst index analysis revealed vegetation recovery trends. This study provides scientific support for ecological management on the QTP.

## Highlights

• LST is the primary driver of vegetation phenology on the QTP
• Effective moisture is a key factor regulating SOS on the QTP
• Surface albedo can characterize human activities
• Future vegetation improvement on the QTP will exceed degradation

## Graphical abstract

**Figure:**
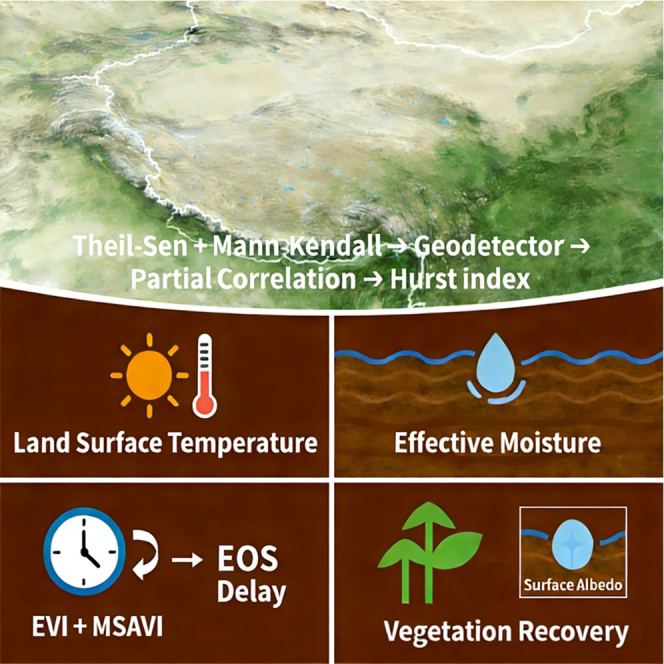

## Introduction

**Figure 1:**
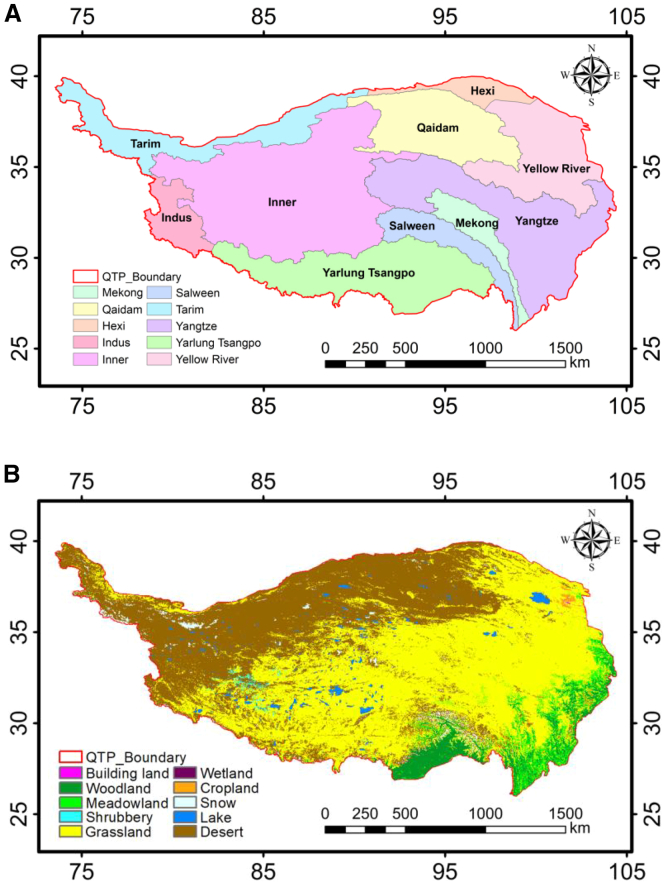
Geographic scope of the study area on the QTP, delineated river basins and land cover types (A) Geographic extent of QTP and delineated river basins in the study area. (B) Geographic extent of QTP in the study area and delineated land cover types. Maps were generated using ArcGIS 10.8.2 (https://www.esri.com/en-us/arcgis/). In (A) and (B), scale bars are standard map elements indicating distance in km.

The Qinghai-Tibet plateau (QTP), referred to as the “roof of the world,” has an average elevation of around 4,000 m. It has both the highest plateau globally and the most extensive expanse of permafrost.^1^^,^^2^^,^^3^^,^^4^ As shown in Figure 1, the distinctive climatic and geographical characteristics of the QTP create a complex and diversified environment.^3^^,^^4^ Simultaneously, it serves as both an “amplifier” and a “sensitive region” for global climate change.^5^^,^^6^ The instability of the QTP ecosystem is evidenced by the ongoing melting of permafrost and the fluctuations in vegetation cover. These elements influence the carbon cycle, the distribution of water resources, and the protection of biodiversity throughout Asia.^7^^,^^8^^,^^9^ Vegetation phenology serves as a crucial indicator of the ecosystem’s reaction to climate change and human activities.^10^ It often denotes the temporal occurrence of cyclical biological activities of flora throughout the annual growth season.^11^^,^^12^ The temporal marker indicating the transition of vegetation from dormancy to active growth, characterized by the emergence of sprouts and foliage, is referred to as the start of the growing season (SOS)^12^^,^^13^; conversely, the temporal marker denoting the shift from active growth to dormancy, marked by wilting and leaf abscission, is termed the end of the growing season (EOS)^12^; and the duration of the transition from active to dormant growth is identified as the length of the growing season (LOS).^12^^,^^14^ By analyzing the SOS, EOS, and LOS, long-term trends in vegetation phenology can be assessed. In recent years, intensification of the greenhouse effect has significantly increased temperatures on the QTP,^1^ subsequently modifying precipitation distribution and water evaporation patterns. Concurrently, human activities such as road construction and grazing expansion continue to alter surface vegetation cover and surface albedo (SA).^15^^,^^16^ Therefore, investigating the drivers influencing vegetation phenology and studying its responses to climate change and human activities are crucial.

Currently, the forefront of vegetation phenology research focuses on multi-source remote sensing data fusion, the differential responses of various vegetation types to climate change, and the spatiotemporal heterogeneity of vegetation phenology and its driving factors.^17^^,^^18^^,^^19^^,^^20^^,^^21^^,^^22^^,^^23^^,^^24^^,^^25^^,^^26^^,^^27^ Despite increasingly diverse research directions, key challenges remain particularly prominent on the QTP. On one hand, regional understanding of the coupling mechanisms between vegetation phenology and hydrothermal variables is insufficient. Meteorological stations on the QTP are densely distributed in the east but sparse in the west, with limited observations in the uninhabited western regions.^3^ The hydrothermal-phenological responses in permafrost areas and the regulatory mechanisms of different vegetation types have yet to be systematically elucidated.^3^^,^^12^^,^^27^^,^^28^^,^^29^^,^^30^ On the other hand, the quantitative precision of human activity impacts on vegetation phenology remains inadequate. Traditional human activity variables often rely on static statistical indicators such as grazing intensity and infrastructure density, failing to capture their dynamic spatiotemporal characteristics.^7^^,^^15^^,^^24^^,^^28^ These issues stem from the significant limitations of traditional hydrothermal variables (e.g., air temperature, precipitation, etc.) and human activity variables on the QTP.^28^^,^^29^^,^^30^^,^^31^^,^^32^^,^^33^^,^^34^^,^^35^^,^^36^^,^^37^^,^^38^^,^^39^ The topographic heterogeneity of the QTP (e.g., high elevation, variable slopes, etc.) makes local observations at individual sites inadequate for representing the actual environmental conditions across broader areas.^5^^,^^15^^,^^16^ In regions with steep elevation gradients, spatial variations in actual surface heat far exceed the air temperature observed at sparse meteorological stations.^3^^,^^18^^,^^40^ Precipitation, subject to topographic redistribution and evapotranspiration differences, cannot be accurately characterized by a single dataset to represent available water for vegetation.^28^^,^^32^ Although traditional variables have supported some important findings, their insufficient spatial representativeness limits the precision of mechanism analysis, particularly in characterizing vegetation phenology responses to microtopographic hydrothermal patterns.^28^^,^^29^^,^^30^^,^^31^^,^^32^^,^^33^^,^^34^^,^^35^^,^^36^^,^^37^^,^^38^^,^^39^ Furthermore, the impact of human activities on vegetation phenology warrants urgent attention. The high-altitude ecosystems of the QTP are fragile and sensitive to human disturbances. In recent years, the intensity and scope of activities such as grazing adjustments and road construction have undergone rapid changes, potentially causing disturbances to local vegetation.^7^^,^^15^^,^^24^^,^^28^^,^^34^^,^^36^^,^^38^ Human activities are not independent of climate but synergistically regulate vegetation phenology with hydrothermal factors by altering the biophysical state of the land surface.^15^^,^^16^ Clarifying their contributions and coupling effects is crucial for precise ecological conservation. Therefore, higher-precision hydrothermal variables and human activity variables must be introduced. This facilitates assessing the current state of the QTP’s ecosystems and formulating corresponding conservation strategies, while also enabling predictions of its future ecological evolution.

The normalized difference vegetation index (NDVI) is a commonly used index in vegetation phenology research,^24^^,^^25^^,^^26^^,^^27^^,^^28^^,^^29^^,^^30^^,^^31^^,^^32^ but it exhibits significant limitations in identifying vegetation phenology on the QTP.^27^^,^^37^ NDVI is highly sensitive to soil background and atmospheric noise, making it difficult to accurately capture phenological signals from sparse vegetation.^27^^,^^37^ Furthermore, the red band saturation effect limits its ability to accurately identify phenological characteristics of dense vegetation.^27^^,^^37^ In previous studies, we employed a combination of the enhanced vegetation index (EVI) and the modified soil-adjusted vegetation index (MSAVI) to capture vegetation phenological characteristics on the QTP.^27^ MSAVI, adjusted for soil effects, is well-suited for the sparse vegetation and early growth stages characteristic on the QTP.^27^ EVI, designed with atmospheric correction, effectively mitigates aerosol interference and alleviates red band saturation, enhancing the identification accuracy of the EOS.^27^^,^^33^^,^^37^ This combination approach overcomes the limitations of NDVI and is more suitable for vegetation phenology studies on the QTP.

Regarding the selection of climate factor data, compared to air temperature, land surface temperature (LST) data can be obtained through satellite remote sensing technology. Its benefits include elevated spatial resolution and temporal continuity, enabling a more precise representation of the real heat distribution experienced by the land surface.^40^ According to relevant studies, LST is closely related to the energy exchange process of vegetation.^41^^,^^42^^,^^43^ For example, changes in LST directly affect vegetation photosynthesis and transpiration.^41^^,^^42^^,^^43^ In regions with notable microtopographic fluctuations, LST may exhibit more dramatic gradient differences compared to air temperature.^44^^,^^45^ This is essential for comprehending vegetation distribution patterns. For instance, under different slope gradients and orientations, LST differences significantly influence SOS and EOS.^46^ Additionally, the moderate resolution imaging spectroradiometer (MODIS) provides long-term continuous LST data from 2000 onwards,^47^ which facilitates analysis of the dynamic response of vegetation phenology on the QTP to thermal environmental changes. Beyond air temperature, accurately assessing vegetation phenology requires calculating effective moisture (EM) based on precipitation and evapotranspiration. This approach enables a more precise evaluation of the impact of moisture on vegetation phenology on the QTP, hence mitigating bias associated with exclusive reliance on precipitation data.

Alongside climate factors, human activities significantly influence vegetation phenology on the QTP. MODIS SA data represent the surface’s ability to reflect solar radiation, reflecting changes in the surface energy balance.^48^^,^^49^ The surface energy balance baseline on the QTP remains relatively stable.^50^ Its hydrothermal variations primarily stem from climatic regulation. Beyond this, human activities such as grazing adjustments, infrastructure expansion, and ecological restoration represent key disturbances driving surface energy changes.^51^ These human activities directly alter surface conditions, subsequently inducing variations in surface albedo.^51^ In comparison to conventional grazing statistics or infrastructure project registration data, SA data presents three significant advantages: firstly, it encompasses a vast observational range, covering the entire plateau area without exclusion; secondly, it boasts a high frequency of updates, enabling timely detection of gradual changes such as pasture degradation; and most importantly, it is derived from satellite remote sensing. This minimizes mistakes that may arise during manual data gathering, enhancing the objectivity and reliability of the data.

In summary, the primary objectives of this study include the following: (1) to elucidate the spatiotemporal evolution characteristics of vegetation phenology and hydrothermal variables (LST, EM, and SA) on the QTP, using the trend analysis and significance stratification rules detailed in Table 1; (2) to identify the core factors and multi-factor coupling mechanisms driving spatiotemporal differentiation in vegetation phenology; (3) to clarify the direct association patterns between vegetation phenology and hydrothermal variables; and (4) to assess the trend persistence in vegetation phenology on the QTP, based on the future trend classification criteria outlined in Table 2, providing scientific support for regional ecosystem conservation and adaptation to climate change.

**Table 1:** Significance stratification based on Theil-Sen trend analysis and Mann-Kendall test

*Slope*_*X*_ and *Z*_*T*_	Significance rating
(1) *Slope*_*X*_ < 0 and (*Z*_*T*_ < −1.96 or *Z*_*T*_ > 1.96)	significant decrease
(2) *Slope*_*X*_ < 0 and (−1.96 ≤ *Z*_*T*_ ≤ −1.64 or 1.64 ≤ *Z*_*T*_ ≤ 1.96)	slightly decrease
(3) (*Slope*_*X*_ < 0 or *Slope*_*X*_ > 0) and −1.64 < *Z*_*T*_ < 1.64	no significant change
(4) *Slope*_*X*_ > 0 and (−1.96 ≤ *Z*_*T*_ ≤ −1.64 or 1.64 ≤ *Z*_*T*_ ≤ 1.96)	slightly increase
(5) *Slope*_*X*_ > 0 and (*Z*_*T*_ < −1.96 or *Z*_*T*_ > 1.96)	significant increase

Note: *Slope*_*X*_ represents the trend slope derived from Theil-Sen analysis, while *Z*_*T*_ denotes the standardized test statistic from the Mann-Kendall test. A *Z*_*T*_ value greater than 1.96 or less than −1.96 indicates statistical significance at the *p* < 0.05 level.

**Table 2:** Classification criteria for future EOS trends based on the H and EOS trend slope

EOS *Slope*	H < 0.4	0.4 < H < 0.6	H > 0.6
*Slope*_*EOS*_ < −0.50	Increase	Uncertain	Decrease
−0.50 < *Slope*_*EOS*_ < 0.50	Stable	Stable	Stable
*Slope*_*EOS*_ > 0.50	Decrease	Uncertain	Increase

Note: *Slope*_*EOS*_ represents the EOS trend slope derived from Theil-Sen analysis. The H assesses trend persistence: H < 0.4 for strong reversal, 0.4 < H < 0.6 for uncertainty, and H > 0.6 for strong continuation.

## Results

### Spatiotemporal variations in vegetation phenology on the QTP from 2001 to 2020

**Figure 2:**
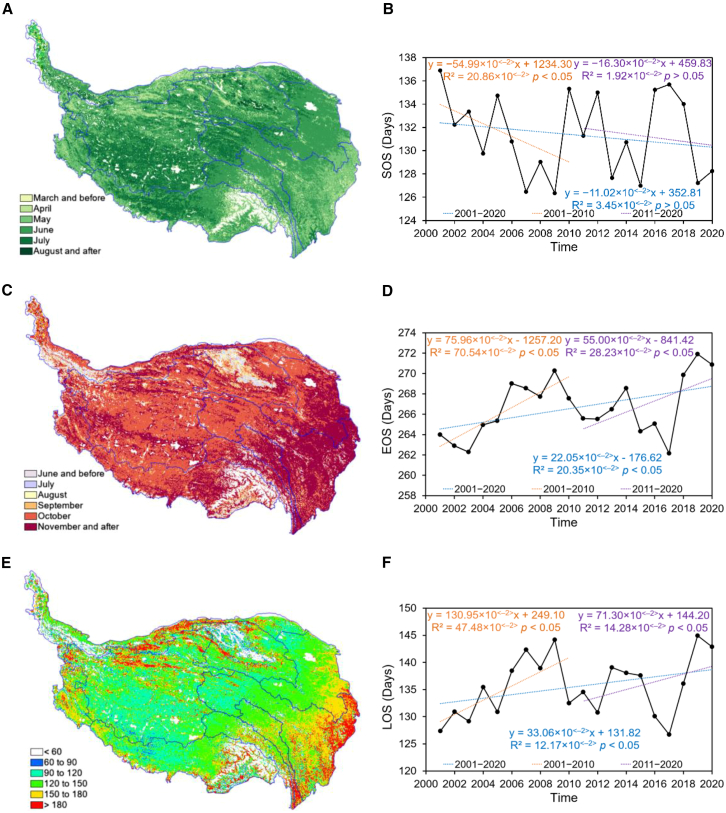
Spatial distribution and interannual trends of vegetation phenology (SOS, EOS, and LOS) on the QTP from 2001 to 2020 Data are represented as annual mean values. Interannual trends were analyzed using the Theil-Sen trend analysis and Mann-Kendall test, with significance defined as *p* < 0.05. (A, C, and E) show the spatial distribution of the annual mean SOS, EOS, and LOS on the QTP from 2001 to 2020. (A and C) indicate the specific months when SOS and EOS occurred, respectively (unit, month). (E) Shows the LOS values (unit, days). (A, C, and E) were generated using ArcGIS 10.8.2 (https://www.esri.com/en-us/arcgis/). (B, D, and F) Show the interannual trends of SOS, EOS, and LOS on the QTP, respectively, the units of SOS, EOS, and LOS trends are d/a. In (B), (D), and (F), *p* values indicate statistical significance from Mann-Kendall tests, with significance defined as *p* < 0.05.

Figure 2A shows that the SOS on the QTP exhibits a trend of gradually advancing from west to east, with an average SOS value of 131.10 days during the period 2001–2020. The proportions of regions with different SOS levels are as follows: March and before (2.27%), April (5.42%), May (24.26%), June (48.91%), July (18.53%), and August and after (0.61%). Regions with SOS occurring in April and before are primarily concentrated in the eastern Yangtze, southern Salween, and southern Mekong. Regions with SOS occurring in July and after are mainly found on the western QTP, northwestern Tarim, and northern Qaidam. In terms of land cover types, regions with SOS occurring in April and before are dominated by meadowland and woodland, while regions with SOS occurring in July and after are dominated by grassland and desert. Figure 2B shows that the SOS on the QTP exhibited a slight advance trend (*p* > 0.05) from 2001 to 2020, with an average annual change rate of 11.02 × 10^<−2>^ d/a. Although both the 2001–2010 and 2011–2020 periods showed an advance trend, the change rate in the first decade was significantly higher than that in the latter decade. From the first decade to the second decade, the advance trend of SOS gradually slowed down.

Figure 2C shows that the EOS on the QTP exhibits a trend of gradual delay from west to east, with an average EOS value of 266.64 days during the period 2001–2020. The proportions of regions with different EOS levels are as follows: June and before (3.52%), July (1.98%), August (3.18%), September (5.44%), October (47.54%), and November and after (38.34%). Regions with EOS occurring in August and before were primarily concentrated in the northwestern Tarim and northern Qaidam. Regions with EOS occurring in November and after were mainly concentrated in the eastern Yangtze and Yellow, southern Salween, and Mekong. In terms of land cover types, regions with EOS occurring in August and before were dominated by grassland and desert, while regions with EOS occurring in November and after were dominated by meadowland and woodland. Figure 2D shows that the EOS on the QTP exhibited a significant delay trend (*p* < 0.05) from 2001 to 2020, with an average annual change rate of 22.05 × 10^<−2>^ d/a. Although both the 2001–2010 and 2011–2020 periods showed a significant delay trend, the change rate in the first decade was higher than that of the latter decade. From the first decade to the second decade, the delay trend of EOS gradually slowed down.

Figure 2E shows that the LOS value on the QTP exhibits a gradual increase from west to east, with an average LOS value of 135.54 days during the period 2001–2020. The proportions of regions with different LOS levels are as follows: less than 60 days (4.53%), 60–90 days (3.80%), 90–120 days (24.19%), 120–150 days (39.68%), 150–180 days (21.62%), and over 180 days (6.17%). Regions with LOS less than 60 days were primarily concentrated in the northwestern Tarim and northern Qaidam. Regions with LOS over 180 days were predominantly distributed in the eastern Yangtze and Yellow, as well as the southern Mekong and Salween. In terms of land cover types, areas with LOS less than 60 days were dominated by grassland and desert, while those with LOS over 180 days were dominated by meadowland and woodland. Figure 2F shows that the LOS on the QTP exhibited a significant increasing trend (*p* < 0.05) from 2001 to 2020, with an average annual change rate of 33.06 × 10^<−2>^ d/a. Although both the 2001–2010 and 2011–2020 periods showed a significant increase trend, the change rate in the first decade was significantly higher than that in the latter decade. From the first decade to the second decade, the increasing trend of LOS exhibited a gradual deceleration.

### Spatiotemporal variations of GLST, GEM, and GSA on the QTP from 2001 to 2020

**Figure 3:**
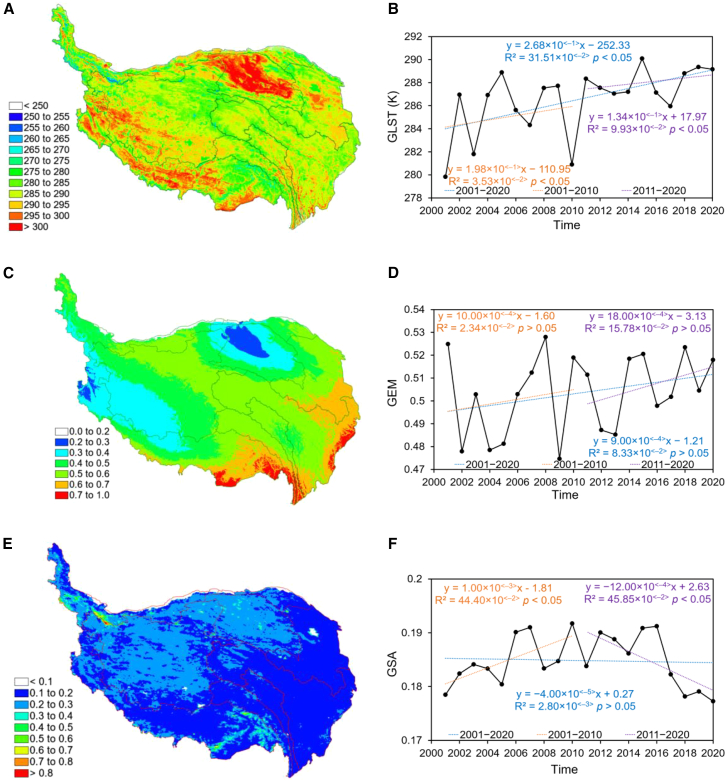
Spatial distribution and interannual trends of GLST, GEM, and GSA on the QTP from 2001 to 2020 Data are represented as annual mean values. Interannual trends were analyzed using the Theil-Sen trend analysis and Mann-Kendall test, with significance defined as *p* < 0.05. (A, C, and E) Show the spatial distribution of the annual mean GLST (unit, K), GEM and GSA on the QTP from 2001 to 2020, respectively. (A, C, and E) were generated using ArcGIS 10.8.2 (https://www.esri.com/en-us/arcgis/). (B, D, and F) Show the interannual trend of GLST (unit, K), GEM and GSA on the QTP from 2001 to 2020, respectively. In (B), (D), and (F), *p* values indicate statistical significance from Mann-Kendall tests, with significance defined as *p* < 0.05.

Figure 3 shows the spatial distribution and interannual trends of average GLST, GEM, and GSA on the QTP from 2001 to 2020. Table 3 presents the characteristics of GLST, GEM, and GSA changes in various river basins on the QTP from 2001 to 2020, based on Theil-Sen trend analysis and Mann-Kendall tests. Table 4 presents the partial correlation analysis results between the interannual trends of hydrothermal variables (GLST and GEM) and vegetation phenology (SOS, EOS, and LOS) on the QTP from 2001 to 2020.

**Table 3:** Characteristics of GLST, GEM, and GSA changes in river basins across the QTP from 2001 to 2020

River basin	Annual change rate of GLST (K/a)	Annual change rate of GEM (/a)	Annual change rate of GSA (/a)
Inner	0.32 × 10^<−2>^	+8.20 × 10^<−4>^	3.50 × 10^<−5>^
Mekong	+2.45 × 10^<−1>∗^	+1.10 × 10^<−3>^	−5.80 × 10^<−4>∗^
Qaidam	+2.72 × 10^<−1>∗^	−7.50 × 10^<−4>∗^	+2.20 × 10^<−4>^
Salween	+2.38 × 10^<−1>∗^	+1.05 × 10^<−3>^	−4.90 × 10^<−4>∗^
Tarim	−0.85 × 10^<−1>^	+6.30 × 10^<−4>^	+6.80 × 10^<−4>∗^
Yangtze	+2.61 × 10^<−1>∗^	+1.30 × 10^<−3>∗^	−6.20 × 10^<−4>∗^
Yellow River	+2.53 × 10^<−1>∗^	+9.50 × 10^<−4>^	−5.50 × 10^<−4>∗^
Yarlung Tsangpo	+2.81 × 10^<−1>∗^	+1.45 × 10^<−3>∗^	−1.20 × 10^<−4>^
Hexi	−0.62 × 10^<−1>^	+7.80 × 10^<−4>^	2.80 × 10^<−5>^
Indus	+1.28 × 10^<−1>^	−6.80 × 10^<−4>∗^	+1.80 × 10^<−4>^

Note: ∗*p* < 0.05 (Theil-Sen trend analysis and Mann-Kendall test), “+”: increase, “−”: decrease, “unsigned”: no significant change.

**Table 4:** Partial correlation analysis results between the interannual trends of hydrothermal variables (GLST and GEM) and vegetation phenology (SOS, EOS, and LOS) on the QTP from 2001 to 2020

Independent variable	Dependent variable	Partial correlation coefficient (r)	Sample size
GLST	SOS	−0.29	20
GLST	EOS	+0.68^∗^	20
GLST	LOS	+0.73^∗^	20
GEM	SOS	−0.32	20
GEM	EOS	−0.35	20
GEM	LOS	+0.61^∗^	20

Note: ∗*p* < 0.05 (partial correlation analysis), “+”: positive correlation, “−”: negative correlation.

From 2001 to 2020, the GLST on the QTP exhibited a significant upward trend (*p* < 0.05), with an average annual warming rate of 2.68 × 10^<−1>^ K/a. Specifically, the increase was 1.98 × 10^<−1>^ K/a from 2001 to 2010 and 1.34 × 10^<−1>^ K/a from 2011 to 2020, indicating a gradual deceleration in the warming trend. The spatial distribution of GLST exhibits pronounced regional heterogeneity: overall, significant increases occurred in the southwest, north, and east, while the northwest and central regions showed more moderate changes. By river basin (a) GLST significantly increased in the Mekong, Qaidam, Salween, Yangtze, Yellow River, and Yarlung Tsangpo basins, with annual rates ranging from 2.38 × 10^<−1>^ to 2.81 × 10^<−1>^ K/a; (b) the GLST in the Tarim and Hexi basins showed a slight decrease, with annual rates of change of 0.85 × 10^<−1>^ K/a and 0.62 × 10^<−1>^ K/a, respectively; and (c) inner regions showed no significant GLST change, with an annual change rate of 0.32 × 10^<−1>^ K/a. Partial correlation analysis revealed: GLST showed a non-significant weak negative correlation with SOS (R = −0.29, *p* > 0.05), a significant positive correlation with EOS (R = 0.68, *p* < 0.05), and a significant positive correlation with LOS (R = 0.73, *p* < 0.05).

From 2001 to 2020, the GEM showed a slight upward trend (*p* > 0.05), with an average annual increase of 9.00 × 10^<−4>^/a. Specifically, the increase was 10.00 × 10^<−4>^/a from 2001 to 2010 and accelerated to 18.00 × 10^<−4>^/a from 2011 to 2020. Spatially, GEM generally increased from north to south and from west to east, with approximately 50% of the regions exhibiting GEM values exceeding 0.50. By river basin (a) GEM significantly increased in the Yangtze and Yarlung Tsangpo basins, with annual change rates of 1.30 × 10^<−3>^ and 1.45 × 10^<−3>^/a, respectively; (b) GEM slightly increased in the Inner, Mekong, Salween, Tarim, Yellow River and Hexi basins, with annual change rates ranging from 6.30 × 10^<−4>^ to 1.10 × 10^<−3>^/a; and (c) GEM significantly decreased in the Qaidam and Indus basins, with annual change rates of 7.50 × 10^<−4>^ and 6.80 × 10^<−4>^/a, respectively. Partial correlation analysis revealed: GEM showed a non-significant weak negative correlation with SOS (R = −0.32, *p* > 0.05), a non-significant weak negative correlation with EOS (R = −0.35, *p* > 0.05), and a significant positive correlation with LOS (R = 0.61, *p* < 0.05).

From 2001 to 2020, the GSA first increased then decreased, showing an overall slight downward trend (*p* > 0.05) with an average annual decrease of 4.00 × 10^<−5>^/a. Spatially, GSA exhibited a decreasing trend from west to east, with approximately 56% of the area having GSA values below 0.20. By river basin (a) GSA in the Tarim Basin showed a significant increase, with an annual change rate of 6.80 × 10^<−4>^/a; (b) GSA in the Qaidam and Indus basins increased slightly, with annual change rates of 2.20 × 10^<−4>^ and 1.80 × 10^<−4>^/a, respectively; (c) GSA in the Mekong, Salween, Yangtze and Yellow River showed significant decreases in GSA, with annual rates of change ranging from 4.90 × 10^<−4>^ to 6.20 × 10^<−4>^/a; (d) the Yarlung Tsangpo experienced a slight decrease in GSA, with an annual rate of change of 2.20 × 10^<−4>^/a; and (e) the GSA of the Inner and Hexi regions showed no significant change, with annual rates of 3.50 × 10^<−5>^ and 2.80 × 10^<−5>^/a, respectively.

### Analysis of driving factors for vegetation phenology on the QTP from 2001 to 2020

**Figure 4:**
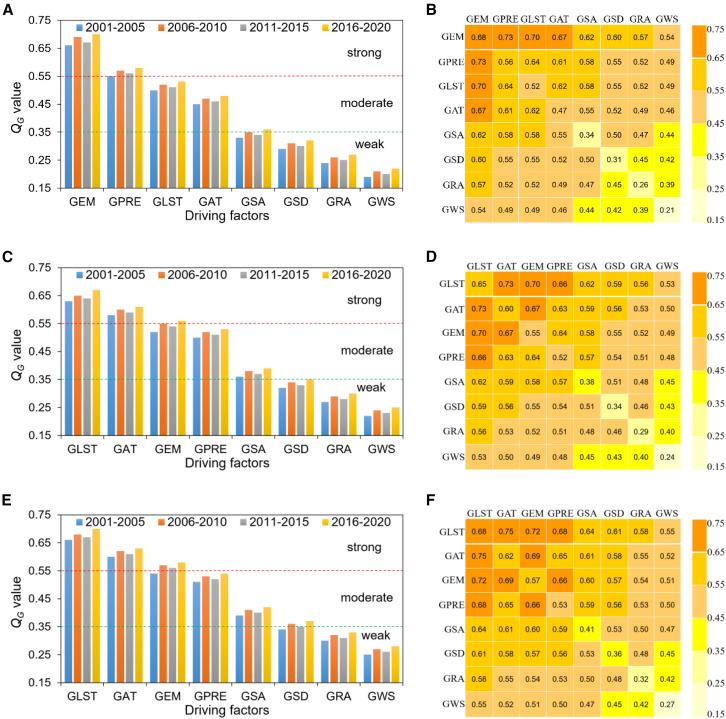
Analysis of single and interacting driving factors for SOS, EOS, and LOS on the QTP from 2001 to 2020 The Geodetector was used to detect the single and interactive driving factors. *Q*_*G*_ > 0.55 (strong), 0.35 ≤ *Q*_*G*_ ≤ 0.55 (moderate), *Q*_*G*_ < 0.35 (weak). (A, C, and E) show the detection results for single driving factors of SOS, EOS and LOS, respectively. *Q*_*G*_ for eight driving factors are displayed in five-year intervals. (B, D, and F) show the detection results for interacting driving factors of SOS, EOS and LOS, respectively. Note, GAT represents growing season air temperature, GSD represents growing season sunshine duration, GRA represents growing season grazing intensity, and GWS represents growing season wind speed.

Figure 4A shows the mean *Q*_*G*_ for each driving factor of SOS, reflecting their contribution to spatial variation in SOS. The primary contributions stem from GEM and GPRE, with GEM making the largest contribution and an explanatory power of 0.68; followed by GPRE with an explanatory power of 0.56. Both demonstrate strong explanatory power for SOS variability (*Q*_*G*_ > 0.55). From a temporal perspective, the *Q*_*G*_ of GEM exhibited a slight upward trend across each cycle, reaching a peak of 0.70 during 2016–2020, a 6.1% increase compared to 2001–2005. The *Q*_*G*_ for GPRE also showed an upward trend, but remained lower than that of GEM across all periods. Figure 4B shows the contribution of 64 pairs of interacting drivers to SOS variation. Among these, the interaction between GEM and GPRE exhibits the highest *Q*_*G*_ (0.73), followed by the interaction between GEM and GLST (0.70).

Figure 4C shows the mean *Q*_*G*_ for each driving factor of EOS, reflecting their contribution to spatial variation in EOS. The primary contributions stem from GLST and GAT, with GLST providing the greatest contribution and an explanatory power of 0.65, followed by GAT with an explanatory power of 0.60. Both demonstrate strong explanatory power for EOS variability (*Q*_*G*_ > 0.55). From a temporal perspective, the *Q*_*G*_ of GLST and GAT exhibited an upward trend across cycles, reaching 0.67 and 0.61 respectively during 2016–2020. Similarly, the *Q*_*G*_ for GEM increased cyclically and remained higher than GPRE across all cycles. Figure 4D shows the contributions of 64 pairs of interacting drivers to EOS variation. Among these, the interaction between GLST and GAT exhibited the highest *Q*_*G*_ (0.73), followed by GLST and GEM (0.70), and GAT and GEM (0.67). The *Q*_*G*_ for the interaction between GLST and GPRE (0.66) exceeded that of the interaction between GEM and GPRE (0.64).

Figure 4E shows the mean *Q*_*G*_ for each driving factor of LOS, reflecting their contribution to spatial variation in LOS. LOS aligns with EOS drivers, with primary contributions concentrated in GLST and GAT. GLST contributes most significantly, explaining 0.68 of variance; GAT follows, explaining 0.62. Both demonstrate strong explanatory power for LOS variability (*Q*_*G*_ > 0.55). Figure 4F shows the contribution of 64 pairs of interacting drivers to LOS variation. The interaction structure aligns with EOS analysis. Among these, the interaction between GLST and GAT exhibited the highest *Q*_*G*_ (0.75), followed by GLST and GEM (0.72), and GAT and GEM (0.69). The *Q*_*G*_ for the interaction between GLST and GPRE (0.68) exceeded that of the interaction between GEM and GPRE (0.66).

### EOS response to changes in GLST and GEM on the QTP during 2001–2020

**Figure 5:**
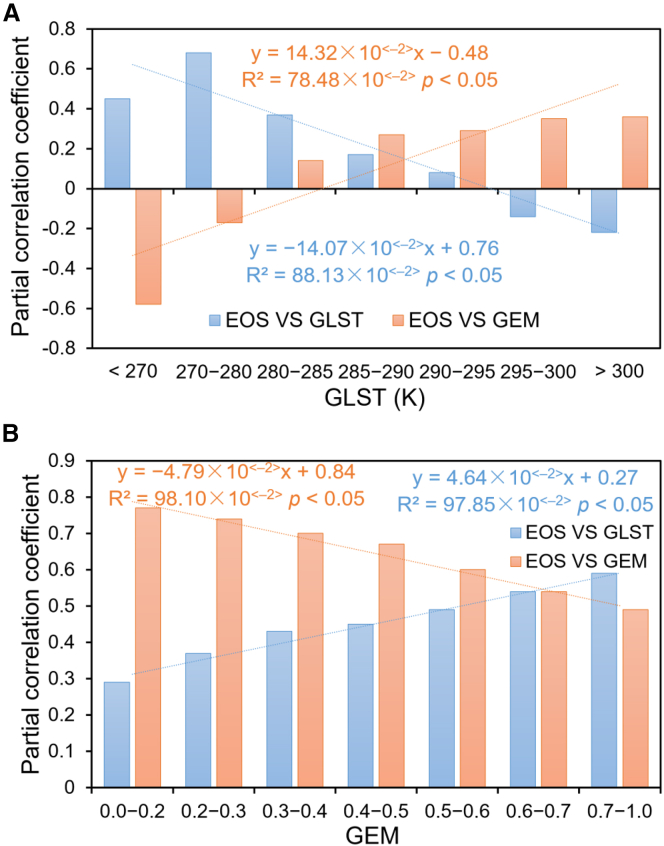
Partial correlation coefficients between EOS and GLST or GEM on the QTP from 2001 to 2020 Partial correlation analysis was used to assess the relationships, with significance defined as *p* < 0.05. (A) Partial correlation coefficients of EOS vs. GLST and EOS vs. GEM at different GLST levels for QTP from 2001*–*2020. (B) Partial correlation coefficients of EOS vs. GLST and EOS vs. GEM at different GEM levels for QTP 2001*–*2020. In (A) and (B), *p* values indicate statistical significance from Mann-Kendall tests, with significance defined as *p* < 0.05.

Figure 5A shows the response of EOS to changes in GLST when GEM remains constant. When GLST is less than 270K, EOS is positively correlated with GLST, while EOS is highly negatively correlated with GEM. As GLST increases, EOS is highly positively correlated with GLST when GLST is between 270K and 280K, while EOS is mildly negatively correlated with GEM. At this time, the ambient temperature was relatively low, and the temperature played a significant role in promoting the growth of vegetation, while moisture inhibited the growth of vegetation. When the GLST reached 280K to 295K, the positive correlation between EOS and GLST gradually decreased, while the positive correlation between EOS and GEM gradually increased. At this time, the ambient temperature was moderate, and the growth of vegetation was affected by the joint influence of water and heat, and the growth of vegetation was more and more strongly affected by water as the temperature increased. When the GLST exceeds 295K, EOS and GLST are negatively correlated, and this negative correlation is gradually increasing with the increase of temperature, while EOS and GEM are positively correlated. At this time, the ambient temperature was high, and as the temperature increased further, it might lead to the decline of the physiological function of the vegetation, and the dependence of the vegetation growth on water became more and more obvious. Figure 5B shows the response of EOS to changes in GEM when GLST remains constant. When GEM is less than 0.20, EOS is highly positively correlated with GEM and EOS is mildly positively correlated with GLST. With the increase of GEM, when GEM is between 0.20 and 0.60, the positive correlation between EOS and GEM is gradually decreasing, while the positive correlation between EOS and GLST is gradually increasing. At this time, the moisture content of the environment is moderate, the growth of vegetation is influenced by water and heat together, and with the increase of moisture content, the dependence of vegetation on water is gradually weakened and the dependence on temperature is gradually strengthened. When GEM was between 0.60 and 0.70, the positive correlation between EOS and both GEM and GLST was almost equal. After GLST exceeded 0.70, the positive correlation between EOS and GLST became stronger than that between EOS and GEM.

### Assessment of future trends in EOS

In this study, H ranged from 0.04 to 0.97 with a mean of 0.52. (a) Further analysis shows that pixels with H < 0.40 accounted for 18.21%, indicating a strongly opposite trend in future EOS changes for these areas. (b) Pixels with H > 0.60 accounted for 45.32%, indicating a strongly consistent trend in future EOS changes for these areas. (c) Pixels with 0.40 < H < 0.60 accounted for 36.47%, implying random future EOS trends in these areas rather than clear consistent or opposite patterns. Overall, the proportion of areas showing strong consistent trends (45.32%) significantly exceeded those with strong opposite trends (18.21%), indicating that the persistent characteristics of future EOS changes in the study area’s vegetation are more pronounced.

**Figure 6:**
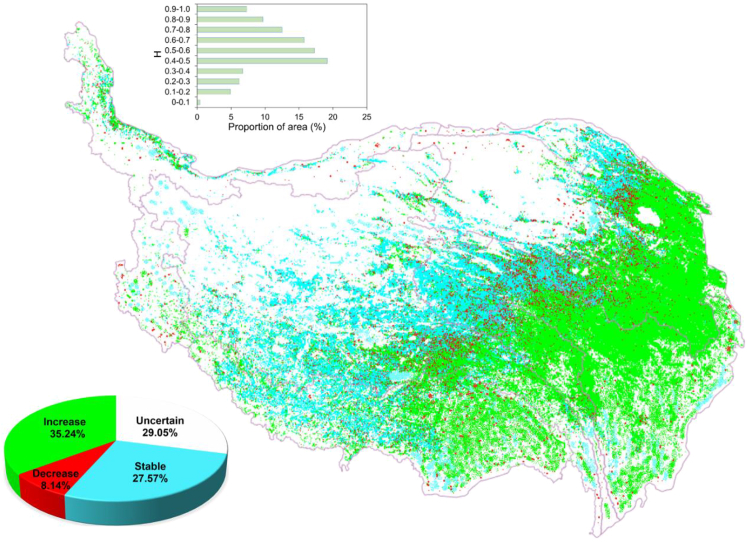
Spatial distribution of assessed future EOS trends and area shares of different EOS trends and H intervals on the QTP from 2001 to 2020 The H was used to assess the persistence of EOS trends, where 0 < H < 0.5 indicates potential trend reversal, 0.5 < H < 1 indicates potential trend continuation, and H = 0.5 indicates a random series. Future EOS trends were classified based on H values and the slopes of historical EOS changes. The map shows the spatial distribution of assessed future EOS trends on the QTP and was generated using ArcGIS 10.8.2 (https://www.esri.com/en-us/arcgis/). The pie chart shows the area share of different future EOS trend categories. The bar chart shows the area proportion of different H intervals across the entire QTP during 2001–2020.

Integrating the H with current EOS change characteristics, Figure 6 illustrates the spatial patterns of projected future EOS trends on the QTP in this study: (a) approximately 35.24% of the region exhibits an EOS delay trend, primarily concentrated in the eastern and southern areas of the QTP. The dominant land cover types in this area are woodland and meadowland; (b) approximately 27.57% of the region exhibits a stable trend, primarily distributed in the central, southwestern, and northeastern parts of the QTP. The dominant land cover types in this area are grassland and shrubbery; (c) approximately 29.05% of the region shows an uncertain trend, mainly distributed in the northern part of the QTP. The dominant land cover types in this area are desert and grassland; and (d) only about 8.14% of the area shows an EOS advance trend, scattered primarily in the central and peripheral regions. The dominant land cover types in these areas are grassland.

## Discussion

### Differences in vegetation and environmental factor trends between permafrost and non-permafrost zones

The QTP, as the world’s highest plateau, exhibits extensive and complex permafrost distribution.^3^ The spatiotemporal variations of its vegetation phenology, GLST, GEM, and GSA are closely linked to permafrost conditions while being significantly regulated by vegetation types.

Unlike previous studies that focused solely on air temperature and precipitation.^30^^,^^32^^,^^33^^,^^34^^,^^35^^,^^36^^,^^37^ This study found that in non-permafrost zones (such as the eastern Yangtze, southern Salween, and Mekong), the active layer is thicker, and GLST and GEM values are higher. The SOS in non-permafrost zones occurs significantly earlier than in stable permafrost zones (approximately 4 months earlier), while the EOS is markedly delayed, resulting in the LOS exceeding 180 days. Vegetation types in non-permafrost zones are predominantly woodland and meadowland. In contrast, stable permafrost zones (such as the northwestern Tarim, northern Qaidam) exhibit not only delayed thawing (lower GLST)^52^ but also limited GEM. Consequently, SOS in stable permafrost areas is generally delayed beyond July, EOS advances before September, and LOS typically falls below 60 days. Vegetation types in stable permafrost zones predominantly consist of grassland. This phenomenon reveals how hydrothermal conditions in permafrost zones constrain vegetation phenological processes.^52^ These findings share commonalities with Zhao et al.’s research on the Northern Hemisphere while also exhibiting regional specificity.^52^ Both studies indicate that SOS onset occurs later in stable permafrost zones than in non-permafrost areas, with the latter exhibiting more favorable hydrothermal conditions.^52^ The difference lies in the shorter LOS observed in the stable permafrost zone on the QTP.^52^ The primary reason for this discrepancy lies in the vegetation phenology extraction methods: this study employed EVI combined with MSAVI, whereas Zhao et al. utilized NDVI. EVI and MSAVI offer greater stability and do not amplify vegetation signals.^27^

In addition to hydrothermal conditions, spatial variations in GSA are also associated with permafrost and vegetation cover types. Higher GSA exceeding 0.30 are observed in high-elevation glacial areas of the northwest and south (predominantly stable permafrost), where land cover is dominated by snow and desert. In the non-permafrost zones of the southeast and east, GSA is lower, ranging from 0.10 to 0.20, with land cover dominated by meadowland and woodland. The high albedo characteristics of glaciers and snow cover, combined with the low vegetation cover of desert vegetation, contribute to the formation of high GSA regions.^53^ Conversely, meadowland and woodland areas with high vegetation cover exhibit significantly reduced GSA.

### Regulation of GEM by active layer thickness in permafrost and differentiation of vegetation types

In addition to precipitation and evapotranspiration, the thickness of the active layer above permafrost is also a key factor influencing the available GEM for vegetation.^54^ On the QTP, grassland is the most widespread vegetation type,^27^ commonly found in both stable and unstable permafrost zones. Grassland typically exhibits moderate GEM (0.40–0.60). In contrast, woodland demonstrates distinct environmental selectivity. Woodland primarily distributed in non-permafrost zones or regions with deep active layers, such as the southern Yarlung Tsangpo and Yangtze. GEM in these areas has risen steadily over the past two decades, providing favorable hydrological support for woodland growth and development. Cropland distribution is more limited, predominantly occurring at the margins of stable permafrost zones, such as in eastern river valleys. Crops are highly sensitive to fluctuations in GEM, and variations in GEM directly impact their yields and phenological processes.

Grassland, woodland, and cropland exhibit distinct responses to long-term changes in GEM. Woodland demonstrates the most sensitive response, with a pronounced upward trend in GEM across their distribution areas. This is attributed to the deeper root systems of woodland and its greater reliance on deep soil moisture regulated by the active layer.^55^ In contrast to woodland, grassland possesses shallower root systems. Thus, their growth is not immediately constrained by declining GEM. Cropland, benefiting from irrigation practices, exhibits a more moderate response to natural moisture fluctuations. It is evident that the thickness of the active layer of permafrost regulates the availability of GEM. This profoundly influences the spatial patterns and dynamic evolution processes of different vegetation types across the QTP.

### Drivers of spatiotemporal differentiation of SOS, EOS, and LOS

In previous study, we identified that the EOS, rather than the SOS, dominates the variation in LOS.^27^ Subsequently, by comparing the attribute differences among vegetation indices EVI, MSAVI and NDVI, and analyzing the correlation between these indices and the interannual change rate of land cover types,^27^ we validated the reliability of this finding. In this study, we further clarified this conclusion from the perspective of driving mechanisms. Between 2001 and 2020, the annual LOS increased by 0.33 days. Delayed EOS contributed 66.67% to this increase, significantly exceeding the contribution from advanced SOS at 33.33%. During interactive driving, the synergistic effect of GLST and GEMG explained vegetation phenology (SOS, EOS and LOS) better than the synergistic effect of GAT and GPRE. Under single-driver conditions, the driving factor sequence for LOS and EOS was consistent, but not for SOS. Specifically, GLST most strongly explained EOS and LOS, while GEM most strongly explained SOS. This indicates that although GEM and GLST jointly govern vegetation growth and development, GLST is the primary driver in high-altitude cold regions like the QTP.^3^ This conclusion contrasts sharply with traditional NDVI-based studies,^24^^,^^30^ which underestimated autumn phenological response sensitivity due to NDVI saturation in cold regions, leading to the conclusion that SOS advancement drives LOS prolongation.^24^^,^^30^ In both previous studies^27^ and the present research, analyses across multiple dimensions have clearly established the dominant role of autumn vegetation phenology (EOS). Additionally, we previously conducted research on permafrost on the QTP.^3^ We discovered that the zero-curtain effect, which characterizes permafrost stability, occurs only in autumn and not in spring.^3^ This series of research findings suggests that future conservation efforts for vegetation and permafrost on the QTP should place greater emphasis on the autumn season. Identifying specific seasonal time frames for implementing these measures can significantly conserve human and material resources.

To clarify the universality and specificity of the aforementioned conclusions, we need to conduct a comparative analysis with existing research. Regarding the driving mechanisms of SOS, this study identifies GEM as the core driver (*Q*_*G*_ = 0.68). This conclusion differs from the findings of Guo et al. in the Qinling mountains,^56^ Wang et al. on the inner Mongolia plateau,^57^ and Liu et al. on the Loess plateau.^58^ These studies all concluded that temperature is the dominant factor for SOS.^56^^,^^57^^,^^58^ The root cause of this discrepancy lies in differing spring moisture conditions across regions. Spring moisture supply is relatively abundant in the Qinling mountains, inner Mongolia plateau, and Loess plateau.^56^^,^^57^^,^^58^ Consequently, temperature becomes the limiting factor for breaking vegetation dormancy and triggering germination. Thus, SOS in these regions is primarily regulated by temperature. For the QTP, extensive cold-tolerant vegetation is distributed here. The germination temperature threshold for this vegetation is approximately 277K.^59^ As shown in Figure 3A, about 85% of the QTP meets this temperature threshold during spring. However, arid zones constitute a significant proportion of the plateau, with relatively scarce precipitation.^39^^,^^60^ Simultaneously, the release of heat stored in permafrost and snowmelt requires prolonged thermal accumulation,^3^^,^^39^^,^^60^ making water availability constraints particularly pronounced. In recent years, the GSA across the QTP has shown an overall downward trend (Figure 3 and Table 3), indicating increased vegetation cover and ecosystem recovery.^51^ Vegetation restoration has further heightened water demand, particularly in high-altitude regions with insufficient spring precipitation, where water supply increasingly relies on snowmelt.^61^ Meltwater formation directly depends on sustained surface heat accumulation,^60^ thereby amplifying GLST’s indirect regulatory role in triggering SOS. Examining spatial heterogeneity (Table 3), GLST continues to rise significantly in regions where GEM shows a declining trend, such as the Indus and Qaidam basins. This indicates that even when moisture conditions remain unchanged or deteriorate, rising temperatures continue to drive vegetation phenological shifts, highlighting temperature’s importance as a consistent cross-regional driver. In regions where GLST and GEM rise synchronously (e.g., Yarlung Tsangpo and Yangtze basins), synergistic effects between the two promote earlier SOS onset and vegetation growth. Thus, although GEM directly constrains SOS, GLST indirectly amplifies its influence by regulating meltwater release. This creates a synergistic mechanism where “temperature unlocks moisture, and moisture determines regreening,” explaining the strengthening trend of temperature’s impact on SOS.

Unlike spring SOS, vegetation has emerged from dormancy by the time autumn enters the EOS phase. The accumulation of photosynthetic products and the leaf senescence process are primarily temperature-regulated.^39^ As shown in Figure 5A, when GLST range between 280K and 295K, the photosynthetic period of vegetation extends, and EOS is delayed accordingly. Conversely, GLST below 280K accelerate vegetation senescence and advance EOS onset. Partial correlation analysis indicates (Table 4) a significant positive correlation between GLST and EOS (R = 0.68), which further strengthens under the context of widespread GLST increases (Table 3). At the regional scale, GLST significantly increased across most basins (particularly pronounced in the Yarlung Tsangpo and Yangtze basins), while GEM exhibited spatial heterogeneity (notably decreasing in the Indus and Qaidam basins), indicating that EOS primarily responds to GLST drivers. Furthermore, the freeze-thaw cycle of permafrost is highly dependent on GLST,^3^ further highlighting its regulatory role. Throughout the entire LOS period, GLST increases simultaneously enhance vegetation physiological activity and GEM accumulation efficiency, forming a positive feedback loop between GLST and GEM (*Q*_*G*_ = 0.72). In summary, against the backdrop of persistent warming on the QTP,^1^^,^^2^ GLST increasingly dominates vegetation phenology: in spring, it indirectly regulates SOS by promoting meltwater accumulation, while in autumn, it directly governs EOS and freeze-thaw processes.

### Differential drivers of GSA on vegetation phenology

GSA serves as a composite factor integrating biophysical signals and human activities.^49^^,^^51^ It exhibits strong explanatory power for QTP vegetation phenology, aligning with the multi-layer regulatory characteristics of the QTP involving “land surface, atmosphere and human activities”.^49^^,^^51^ From a driving mechanism perspective, warming at the climatic level triggers permafrost degradation, altering surface roughness and soil moisture conditions, thereby directly reshaping the baseline level of GSA.^62^ Meanwhile, human activities such as overgrazing and increased construction at the anthropogenic level further amplify the dynamic fluctuations of GSA.^63^ Additionally, spatial differentiation in QTP hydrothermal conditions shaped the differential driving patterns of GSA on vegetation phenology: (a) in woodland regions of the southeastern Yangtze River Basin, GSA reflects the regulatory role of ecological restoration in surface energy exchange.^24^ It synergistically interacts with GEM to jointly drive phenological changes in woodlands; (b) in the grasslands of the central-northern Qaidam Basin, GSA reflects the disturbance of surface conditions caused by grazing;^63^ its regulation of surface energy simultaneously promotes meltwater recharge, thereby accelerating grassland phenological processes; and (c) in the northeastern cropland regions of the Yellow river basin, hydrothermal conditions remain relatively stable. GSA precisely regulates key phenological stages by mapping the impacts of human management activities.^63^ These differentiated patterns also validate GSA’s adaptability across diverse vegetation and environmental combinations on the QTP.

Based on GSA’s differentiated responses across various vegetation and environmental combinations, it offers valuable perspectives for vegetation phenology research on the QTP. GSA clarifies the climate adaptation directions for each region: southeastern woodlands should focus on mitigating potential drought risks under fluctuating precipitation patterns; central-northern grasslands need to balance permafrost conservation with the ecological benefits of moderate warming; and eastern river valley farmlands should address growth stresses caused by rising temperatures through breeding heat-tolerant crops.

### Exploring future trends in vegetation phenology

This study extracts the EOS for vegetation phenology on the QTP using EVI and MSAVI, and analyzes future trends using the H. The results differ significantly from previous studies: In this study, 45.32% of regions show a strongly consistent trend (H > 0.6), while only 36.47% exhibit an uncertain trend (0.4 < H < 0.6); across the QTP, 35.24% of areas exhibit an increasing EOS trend, while only 8.14% show a decreasing trend. Previous studies, primarily based on NDVI or its derivative vegetation cover to analyze future vegetation trends,^28^^,^^64^^,^^65^ reported over 60% uncertainty in trend identification, less than 6% strong consistency, and generally concluded that “vegetation degradation exceeds improvement”.^28^^,^^64^^,^^65^

This discrepancy fundamentally stems from the inherent differences between EVI, MSAVI, and NDVI. Their respective capabilities in handling disturbance factors directly determine trend identification accuracy. NDVI lacks soil background adjustment and atmospheric aerosol correction components, making it susceptible to interference from soil reflectance and atmospheric scattering in sparsely vegetated regions like the QTP.^66^ Its time-series oscillation amplitude (−0.50 to 1.00) is significantly larger than that of MSAVI and EVI (−0.30 to 0.90).^27^ In contrast, EVI incorporates blue band data and atmospheric correction coefficients to reduce atmospheric interference,^67^ while MSAVI employs soil adjustment factors to mitigate soil effects, making it more sensitive to early-stage vegetation and low-coverage areas.^68^ The correlation coefficients between EVI/MSAVI and interannual change rates for primary vegetation types like grasslands and meadows reached 0.78 and 0.82, respectively. This significantly exceeds NDVI’s values of 0.39 and 0.41.^27^ Consequently, the vegetation information obtained in this study is more accurate, reducing uncertainty in trend assessments.

This study captures the trend of “greater vegetation improvement than degradation,” which is closely linked to proactive human interventions: grazing restrictions, ecological conservation red line policies, and afforestation along desert margins have significantly enhanced the positive continuity of natural recovery processes.^69^^,^^70^^,^^71^^,^^72^^,^^73^ For instance, in the central-eastern QTP, human conservation measures directly promoted vegetation restoration, reinforcing the positive trajectory of natural processes^73^; In adjacent regions like the Taklamakan Desert, afforestation and wetland restoration practices similarly demonstrate the effectiveness of human interventions in curbing desert expansion and promoting vegetation recovery.^74^ Furthermore, the significant decline in GSA over the past decade, as shown in Figure 3F, further corroborates the positive effects of human activities on the QTP. Collectively, this evidence supports the validity of this study’s conclusions.

Building upon the preceding systematic analysis of permafrost zone variations, GEM regulatory mechanisms, vegetation phenological drivers, and the GSA effect, this study distills the key findings: (a) non-permafrost zones exhibit superior hydrothermal conditions with an LOS exceeding 180 days, whereas stable permafrost zones face constraints from low temperatures and water scarcity, resulting in an LOS of less than 60 days; (b) autumn EOS serves as the dominant factor regulating LOS, while GLST is the core driver of vegetation phenological processes; and (c) H analysis based on EOS indicates that future vegetation improvement on the QTP will outweigh degradation, confirming the positive effects of regional ecological conservation policies. Based on these findings, three measures are proposed for regional ecological conservation and sustainable management: first, considering permafrost differentiation characteristics, maintain high-coverage vegetation patterns like woodlands and meadowlands in the non-permafrost areas of the southeast, while strictly controlling grassland disturbance in the stable permafrost zones of the north. Second, focusing on EOS’s dominant role, designate autumn as the key monitoring period for permafrost and vegetation phenology, and synchronously adjust ecological management windows. Third, based on GSA characteristics, promote afforestation and wetland restoration in vegetation degradation fringe zones to enhance precision in vegetation recovery within high-altitude cold regions.

### Limitations of the study

The unique environment of the QTP also imposes constraints on GSA application. First, during the spring thaw period in permafrost regions, GSA is subject to overlapping interference from freeze-thaw physical processes and vegetation recovery, making it difficult to isolate the contribution of human activities.^37^ Secondly, autumn clouds and snow cover in the central and northern regions reduce the accuracy of GSA optical remote sensing inversion.^54^ Additionally, GSA in sparsely vegetated areas is susceptible to soil background interference, while GSA in high-canopy forested areas may mask minor human disturbances.^54^ To address these limitations, future studies may consider utilizing microwave data to compensate for the shortcomings of optical remote sensing in GSA inversion and application.

## Resource availability

### Lead contact

Requests for further information and resources should be directed to and will be fulfilled by the lead contact, Zhijian Zhao (zhaozhijian1222@126.com).

### Materials availability

This study did not generate unique research materials.

### Data and code availability

• The research data that were utilized in this investigation are kept in two different databases, namely LP DAAC and TPDC. This study makes use of the following datasets, which can be accessible through the following links:
  ○ LP DAAC: https://doi.org/10.5067/MODIS/MOD09A1.006
  ○ LP DAAC: https://doi.org/10.5067/MODIS/MOD11A2.061
  ○ LP DAAC: https://doi.org/10.5067/MODIS/MOD16A2.006
  ○ LP DAAC: https://doi.org/10.5067/MODIS/MCD43A3.006
  ○ LP DAAC: https://doi.org/10.5067/MODIS/MCD12Q1.006
  ○ TPDC: https://doi.org/10.11888/Atmos.tpdc.300470
  ○ TPDC: https://doi.org/10.11888/Ecolo.tpdc.271513
• This study does not report original code.
• Any additional information required to reanalyze the data reported in this article is available from the lead contact upon request.

## Acknowledgments

Each of the following organizations provided financial support for this study: the Science and Technology Research Project of the 10.13039/501100009102Education Department of Jiangxi Province (grant no. GJJ2502105), the 10.13039/501100001809National Natural Science Foundation of China (grant no. 42330108), the Science and Technology Research Project of the 10.13039/501100009102Education Department of Jiangxi Province (grant no. GJJ2402102) and the 10.13039/501100001809National Natural Science Foundation of China (grant no. 62441112).

## Author contributions

Conceptualization, Z.Z. and H.L.; methodology, Z.Z., L. Wang, and M.H.; software, Z.Z. and L. Wu; validation, Z.Z. and M.H.; formal analysis, Z.Z., L.T., and L. Wang; investigation, Z.Z. and H.L.; resources, Z.Z. and L. Wu; data curation, Z.Z., L.T., and T.Y.; writing – original draft preparation, Z.Z., M.H., and X.X.; writing – review and editing, Z.Z. and L. Wang; visualization, Z.Z. and T.Y.; supervision, Z.Z. and X.X.; project administration, Z.Z., H.L., and L. Wang; funding acquisition, Z.Z., L. Wu, and H.L. All authors have read and agreed to the published version of the manuscript.

## Declaration of interests

The authors declare no competing interests.

## Declaration of generative AI and AI-assisted technologies in the writing process

During the preparation of this work, the authors did not use any generative AI or AI-assisted technologies that generate original content. All content is the original work of the authors, who take full responsibility for the accuracy.

## STAR★Methods

### Key resources table

**Table:** 

REAGENT or RESOURCE	SOURCE	IDENTIFIER
**Deposited data**		

MOD09A1 data	LP DAAC	https://doi.org/10.5067/MODIS/MOD09A1.006
MOD11A2 data	LP DAAC	https://doi.org/10.5067/MODIS/MOD11A2.061
MOD16A2 data	LP DAAC	https://doi.org/10.5067/MODIS/MOD16A2.006
MCD43A3 data	LP DAAC	https://doi.org/10.5067/MODIS/MCD43A3.006
MCD12Q1 data	LP DAAC	https://doi.org/10.5067/MODIS/MCD12Q1.006
Meteorological data	TPDC	https://doi.org/10.11888/Atmos.tpdc.300470
Grazing intensity data	TPDC	https://doi.org/10.11888/Ecolo.tpdc.271513

**Software and algorithms**		

ArcGIS 10.8.2	Esri	https://www.esri.com/en-us/arcgis/
MRT	LP DAAC	https://lpdaac.usgs.gov/tools
MATLAB 2022b	MathWorks	https://www.mathworks.com/products/matlab.html
Geodetector	State Key Laboratory of Resources and Environment Information System, Institute of Geographic Sciences and Natural Resources Research, Chinese Academy of Sciences	http://www.geodetector.cn/

### Experimental model and study participant details

This study involves remote sensing observation and spatial analysis research and does not involve experimental models, animal/human subjects, microbial strains, cell lines, or primary cell cultures. Therefore, this section does not apply.

### Method details

#### Study area

The QTP is situated in southwestern China. It extends from 73°19′ to 104°47′ E and from 26°00′ to 39°47′ N. The QTP stretches approximately 1,500 km from north to south and nearly 3,000 km from east to west. The QTP is the world’s most topographically complex plateau.^1^^,^^3^^,^^4^ Its immense elevation variations and vast east-west span have created significant latitudinal and vertical zonation in hydrothermal conditions.^1^^,^^3^^,^^15^ This provides a natural laboratory for studying the spatiotemporal evolution of vegetation phenology and its driving mechanisms. The landscape of the QTP is characterized by an interwoven pattern of plateau surfaces, towering mountain ranges, and intermontane basins. This topographic configuration profoundly influences the distribution of surface energy and moisture.^1^^,^^3^^,^^4^^,^^30^^,^^34^ This results in high spatial heterogeneity in key hydrothermal variables such as LST, EM, and SA.^1^^,^^3^^,^^5^^,^^30^ The climate of the QTP exhibits typical alpine characteristics, with most regions having an annual average temperature below 0°C.^3^^,^^28^ Driven by climatic gradients, vegetation patterns successively transition from southeast to northwest through woodland, meadowland, shrubbery, grassland and ultimately deserts.^3^^,^^4^^,^^28^^,^^34^ This distinct zonation pattern provides an ideal spatial sequence for investigating the response of vegetation phenology to hydrothermal conditions. Furthermore, as the “water tower of Asia” and a region with extensive permafrost distribution, the QTP exhibits tight coupling among permafrost, vegetation, and hydrological processes.^3^^,^^4^^,^^5^^,^^27^^,^^28^ Permafrost degradation not only directly alters soil hydrothermal conditions but may also indirectly regulate vegetation phenology by influencing SA and evapotranspiration.^1^^,^^3^^,^^5^ In summary, the QTP was selected as the study area.

#### Data sources and pre-processing

A total of seven types of data were used in this study. (a) The first is the MOD09A1 data (LP DAAC: https://doi.org/10.5067/MODIS/MOD09A1.006 (accessed on 15 November 2024)), which provides 500 m resolution bands 1-7 in an 8-day grid of sinusoidal projection. Since MOD09A1 data are extracted based on the conditions of cloud-free, high observational coverage, and low viewing angle, each of its image elements contains 8 days of the best L2G observations. In this study, MOD09A1 data are used to analyze the EVI and MSAVI of the QTP. (b) The second is the MOD11A2 data (LP DAAC: https://doi.org/10.5067/MODIS/MOD11A2.061 (accessed on 15 November 2024)), which has a grid of 1,200 × 1,200 km with a spatial resolution of 1 km, and is provided every 8 days, including diurnal and nocturnal LST. MOD11A2 data provide the associated observation time, quality control assessment, clear sky coverage, viewing zenith angle, and radiance in bands 31 and 32. The data has undergone Terra MODIS infrared band crosstalk correction and meets Stage 2 validation standards, fulfilling research requirements. In this study, MOD11A2 data are used to resolve the LST on the QTP. (c) The third is the MOD16A2 data (LP DAAC: https://doi.org/10.5067/MODIS/MOD16A2.006 (accessed on 15 November 2024)), which has a spatial resolution of 500 m. The pixel values of evapotranspiration are averaged every 8 days, except for the last collection period of each year (the last collection period is 5 or 6 days depending on the year). In this study, MOD16A2 data were used to resolve evapotranspiration on the QTP. (d) The fourth is the MCD43A3 data (LP DAAC: https://doi.org/10.5067/MODIS/MCD43A3.006 (accessed on 15 November 2024)), which is in ortho-selected projection with a spatial resolution of 500 m. The MCD43A3 data is retrieved once a day and represents the best Bidirectional Reflectance Distribution Function (BRDF) based on 16 days of input data. MCD43A3 data is a combined product of Terra and Aqua, which can provide directed dark sky albedo and daytime albedo. In this study, MCD43A3 data were used to resolve SA on the QTP. (e) The fifth is the MCD12Q1 data (LP DAAC: https://doi.org/10.5067/MODIS/MCD12Q1.006 (accessed on 15 November 2024)), which has a spatial resolution of 500 m and is a combined product of Terra and Aqua, provided once a year. This data contains five different land cover classification schemes, of which the International Geosphere-Biosphere Programme (IGBP) was used in this study to resolve the land cover classification of the QTP. (f) The sixth is meteorological data, including indicators such as air temperature, precipitation, sunshine duration and wind speed (TPDC: https://doi.org/10.11888/Atmos.tpdc.300470 (accessed on 15 November 2024)). This dataset is provided by the National Tibetan Plateau Data Center. Observed meteorological data spans from 1980 to 2020, with an original temporal resolution at the daily scale. This dataset achieves a spatial resolution of 500 m through Anusplin interpolation software, utilizing the World Geodetic System 1984 (WGS84) coordinate system. This dataset was utilized in this study to investigate the driving mechanisms of vegetation phenology. (g) The seventh is the grazing intensity dataset (TPDC: https://doi.org/10.11888/Ecolo.tpdc.271513 (accessed on 15 November 2024)). This dataset is provided by the National Tibetan Plateau Data Center. The data is compiled based on actual carrying capacities across seven core pastoral areas in eastern and southwestern parts of the QTP. Through spatial modeling, it generates raster data for grazing intensity (sheep units, MU/km^2^). The temporal coverage spans 2000–2019, with a native temporal resolution of daily and a spatial resolution of 250 m. This dataset is primarily utilized in analyzing dominant factors and multi-factor interactions influencing spatiotemporal variation in vegetation phenology.

For the first six types of data mentioned above, the study period used in this study is 2001–2020. For the seventh type of data, the study period used in this study is 2001–2019. Pre-processing was performed on these seven datasets. First, the MODIS Reprojection Tool (MRT) was used for data stitching and resampling, uniformly resampling the spatial resolution of all datasets to 500 m. Regarding temporal resolution, except for the MCD12Q1 data used for land cover classification analysis, which is on an annual scale, all data were aggregated to an 8-day scale to align with the analysis requirements for vegetation phenology dynamics. Subsequently, all data were uniformly projected into the WGS84 coordinate system. Finally, data were clipped according to the vector boundaries of the QTP.

Figure 1A was produced using the Hydrology Tools of ArcGIS 10.8.2, derived on an analysis of river distribution across the QTP. It utilizes the Shuttle Radar Topography Mission (SRTM) 30 m resolution Digital Elevation Model (DEM) as the foundational topography data, using hydrological modeling processes like watershed extraction and river network generation. The QTP is divided into 10 river basins, including Inner, Mekong, Qaidam, Salween, Tarim, Yangtze, Yellow River, Yarlung Tsangpo, Hexi and Indus. Nourished by many rivers, the QTP has formed an extremely rich variety of land cover types. Figure 1B was generated by analyzing actual land cover data for the QTP using ArcGIS 10.8.2. The land cover data originates from the MODIS MCD12Q1 2014 land cover product (LP DAAC: https://doi.org/10.5067/MODIS/MCD12Q1.006 (accessed on 15 November 2024)), featuring a spatial resolution of 500 m and annual availability. Based on this data and the actual land cover conditions of the QTP, the region has been classified into 10 landcover types: desert, lake, snow, cropland, wetland, grassland, shrubbery, meadowland, woodland, building land.

#### Extraction of vegetation phenology on the QTP during 2001–2020

In previous studies, we demonstrated that EVI and MSAVI are more reliable than NDVI for extracting vegetation phenology on the QTP.^27^ Therefore, this study employs a combined approach using EVI and MSAVI to extract vegetation phenology. The processing steps are outlined as follows:

(a) Preprocessed 8-day time series data of EVI and MSAVI from 2001 to 2020 to eliminate snow cover interference. (b) Reconstructed EVI and MSAVI time series data using the Savitzky-Golay filtering method to remove random noise.^75^^,^^76^ (c) Calculate the annual averages of EVI and MSAVI during the growing season (May to October) from 2001 to 2020. (d) Employ the dynamic threshold method^27^^,^^77^^,^^78^ to extract vegetation phenological, dividing them into one experimental group and three control groups. For the experimental group, EOS was extracted based on EVI, and SOS based on MSAVI; for the control groups, SOS and EOS were extracted based on each vegetation index (EVI, MSAVI, and NDVI), respectively. The LOS for both experimental and control groups was calculated as the difference between their EOS and SOS values.

The performance of this combined approach was validated in three aspects: (a) Correlation analysis: The correlation coefficients between MSAVI or EVI and the interannual change rates of major vegetation types such as grassland, meadowland and woodland reached 0.78, 0.82 and 0.75, respectively, significantly higher than the corresponding values of 0.39, 0.41, and 0.67 for NDVI. (b) Comparative validation: The correlation between LOS obtained by the experimental group and land cover change was 0.67, higher than the values of 0.63, 0.65, and 0.55 in the control group. (c) Consistency test: MSAVI and EVI demonstrated superior correlation in mean and maximum values, with more stable temporal variations. The LOS increase magnitude and spatial extent derived from the combination method better aligned with the actual ecological conditions of the QTP.

Detailed workflows and algorithms are documented in our previous study.^27^

#### Extraction of GEM, GLST and GSA on the QTP during 2001–2020

The hydrothermal data used in this study were EM and LST, respectively. EM combines the effects of precipitation and evapotranspiration and is calculated as:

(Equation 1) $$\text{GEM}=\frac{\text{GPRE}-\text{GET}}{\text{GPRE}}$$

where GPRE is the precipitation for the growing season each year. GET is the evapotranspiration of the growing season each year. GEM is the effective moisture index of the growing season each year, and the value ranges from 0 to 1. GEM close to 0 represents poor moisture conditions, and on the contrary, when GEM is close to 1, it represents good moisture conditions.

The LST data used in this study represent the average values of day and night LST after quality control. The growing season LST (GLST) refers to the average LST values recorded from May to October each year.

In addition to EM and LST, MODIS SA data were used in this study to characterize human activities on the QTP. The calculation method of shortwave albedo proposed by Liang^79^^,^^80^ was used in extracting the SA:

(Equation 2) $$B_{short}=0.160B_{1}+0.291B_{2}+0.243B_{3}+0.116B_{4}+0.112B_{5}+0.081B_{7}-0.0015$$

where *B*_*1*_, *B*_*2*_, *B*_*3*_, *B*_*4*_, *B*_*5*_, and *B*_*7*_ correspond to bands 1, 2, 3, 4, 5 and 7 in the MCD43A3 data, respectively, while *B*_*short*_ denotes the shortwave albedo. In this study, SA was calculated by averaging diurnal SA, and GSA was the average of SA from May to October each year.

Additionally, the applicability of the empirical coefficients in Equation 2 to the QTP is explained as follows: These coefficients were calibrated using measured data from over 30 global radiation observation stations, covering both high-altitude and arid zones. The calibration process encompassed six primary land surface types: woodland, grassland, cropland, desert, snow and lake.^79^^,^^80^ This closely matches the predominant surface types on the QTP. Liang’s global validation results indicate that the RMSE for shortwave albedo calculations across different land surface types does not exceed 0.03 ^57^. This meets the accuracy requirements for SA analysis in this study.

#### Analysis and testing of trends

In this study, a combination of Theil-Sen trend analysis and Mann-Kendall test^81^^,^^82^ was used to analyze the trends of various factors on the QTP during 2001–2020. These factors include: SOS, EOS, LOS, GEM, GLST and GSA, etc. Theil-Sen trend analysis is highly resistant to interference and does not require a strict distribution of data. The formula is as follows:

(Equation 3) $${Slope}_{X}=Med\left(\frac{X_{J}-X_{I}}{J-I}\right)\;2001\leq I<J\leq 2020$$

where *J* and *I* denote the intervals of the time series, and *X*_*J*_ and *X*_*I*_ denote the values of *X* corresponding to *J* and *I*, respectively. *Slope*_*X*_ denotes the trend of the variable in the given time series, with a downward trend when *Slope*_*X*_ < 0 and an upward trend when *Slope*_*X*_ > 0.

The Mann-Kendall test is nonparametric and determines whether a trend is significant by calculating a statistic for a consistent trend. It is generally used in conjunction with Theil-Sen trend analysis. Its evaluation method is as follows:

Firstly, the test statistic *S*_*T*_ is calculated for the data series with the formula:

(Equation 4) $$S_{T}=\sum_{I=1}^{K-1}\sum_{J=I+1}^{K}Sgn\left(X_{J}-X_{I}\right)$$

(Equation 5) $$Sgn\left(\varphi \right)=\left\{ \begin{array}{c} -1,\varphi <0\\ 0,\varphi =0\\ 1,\varphi >0 \end{array}\right.$$

(Equation 6) $$Var\left(S_{T}\right)=\frac{K\left(K-1\right)\left(2K+5\right)}{18}$$

where the set length of the data sequence *X* is *K*, *X*_*J*_ and *X*_*I*_ are the sample data in the set *X*. *Sgn* is the sign function used to determine the sign of *(X*_*J*_ – *X*_*I*_*)* and *Var(S*_*T*_*)* is the variance. After that, the standardized test statistic *Z*_*T*_ was calculated with the formula:

(Equation 7) $$Z_{T}=\left\{ \begin{array}{c} \left(S_{T}+1\right)/\sqrt{Var\left(S_{T}\right)\;},S_{T}<0\\ 0,S_{T}=0\\ \left(S_{T}-1\right)/\sqrt{Var\left(S_{T}\right)\;},S_{T}>0 \end{array}\right.$$

When *Z*_*T*_ > 0 or *Z*_*T*_ < 0, the data series is trending upward or downward. When the *Z*_*T*_ falls within a specific range, the trend of the data can be effectively judged. For example, if the significance level *β* is 0.05, when the statistic reaches 1.96, it indicates that the significance of the result can be confirmed at a 95% confidence level. Similarly, a 90% significance test was passed when *β* = 0.10 and the *Z*_*T*_ statistic was 1.64. Therefore, based on the combination of Theil-Sen trend analysis and Mann-Kendall test the significance determination rule can be derived as shown in Table 1.

#### Driving force analysis based on geodetector

Geodetector is based on the principle of spatial hierarchical heterogeneity.^83^^,^^84^^,^^85^ The primary role is to measure the explanatory capacity of independent variables on dependent variables using the *Q*_*G*_ statistic, computed as follows:

(Equation 8) $$Q_{G}=1-\frac{\sum_{U=1}^{W}D_{U}\delta_{U}^{2}}{D\delta^{2}}$$

where *D* represents the total sample size in the study area, *δ*^2^ denotes the total variance of the variables, *W* indicates the number of strata for the independent variables, *D*_*U*_ signifies the sample size in the Uth stratum, and $\delta_{U}^{2}$ represents the variance of the dependent variable in the Uth stratum. The *Q*_*G*_ value ranges from 0 to 1. In this study, it is classified as follows: *Q*_*G*_ > 0.55 (strong), 0.35 ≤ *Q*_*G*_ ≤ 0.55 (moderate), *Q*_*G*_ < 0.35 (weak). Furthermore, the significance of *Q*_*G*_ can be tested using a noncentral F-distribution to exclude random error interference.^83^^,^^84^^,^^85^

This model requires no predetermined variable associations and is resistant to multicollinearity. It may discern core driving elements via factor recognition and evaluate synergistic effects across factors through interaction detection.^83^^,^^84^^,^^85^ This study employs the model to investigate the spatiotemporal variation mechanisms of vegetation phenology (SOS, EOS, and LOS) on the QTP, elucidating the influence pathways and driving forces of various factors. This analysis selected eight key independent variables: GEM, GLST, GSA, precipitation, air temperature, sunshine duration, wind speed, and grazing intensity. Regarding the data time frame, grazing intensity data spans the growing seasons from 2001 to 2019. For the remaining seven variables, the reference time span is set to the growing seasons from 2001 to 2020.

Since the Geodetector requires the independent variable to be categorical, the eight continuous variables involved in this study must first undergo discretization. The quantile method was employed for this discretization.^84^^,^^86^ This approach ensures relatively uniform sample size distribution across each stratum, reducing *Q*_*G*_ value bias caused by excessive variation in stratum-specific sample sizes.^84^^,^^86^ The specific operation was implemented using the Reclassify tool in ArcGIS 10.8.2 software. Each continuous variable was divided into five equal quantiles: 0–0.2, 0.2–0.4, 0.4–0.6, 0.6–0.8, and 0.8–1.0. Each stratum contained approximately 20% of the total sample size. This ensured the stratified results met the analytical requirements of the geographic detector.

#### Correlation analysis between EOS and GLST, GEM

Partial correlation analysis quantifies the mutual correlation between two variables by controlling one or more variables. Furthermore, partial correlation analysis eliminates the influence of other variables in a multivariate system.

In this study, the correlation between EOS, GLST and GEM was evaluated by partial correlation analysis. The algorithm for the partial correlation coefficient is:

(Equation 9) $$R_{\left(VL|M\right)}=\frac{R_{VL}-R_{VM}\times R_{LM}}{\sqrt{\left(1-R_{VM}^{2}\right)\times \left(1-R_{LM}^{2}\right)}}$$

(Equation 10) $$R_{\left(VM|L\right)}=\frac{R_{VM}-R_{VL}\times R_{LM}}{\sqrt{\left(1-R_{VL}^{2}\right)\times \left(1-R_{LM}^{2}\right)}}$$

where *R*_*VL|M*_ is the partial correlation coefficient between EOS and GLST, and *R*_*VM|L*_ is the partial correlation coefficient between EOS and GEM. *R*_*VL*_, *R*_*VM*_, and *R*_*LM*_ are the correlation coefficients between EOS and GLST, EOS and GEM, and LST and GEM, respectively.

#### Sustainability assessment of trends in EOS changes

The Hurst index (H) is an important metric in statistics, which is mainly used to assess the trend persistence or inverse persistence of a time series.^87^ It should be clarified that the H is not intended for quantitative forecasting of future scenarios. Instead, it analyzes the self-similarity of historical time series to determine the tendency for existing trends to persist or reverse. The value of H ranges from 0 to 1. When H = 0.50, the sequence exhibits random characteristics, making it impossible to determine whether the existing trend will persist or reverse. When 0.50 < H < 1, the existing trend in the sequence is likely to persist (exhibiting positive correlation characteristics). Conversely, when 0 < H < 0.50, the existing trend in the sequence is likely to reverse (exhibiting negative correlation characteristics). This study employs the H value to assess the persistence tendency of the current change trend in EOS.

In this study, the H was calculated using Rescaled Range Analysis (R/S analysis),^87^ with specific steps tailored to the EOS time series (*P*):

a. EOS time series preprocessing: The 3σ rule is applied to remove outliers from the EOS sequence, ensuring the stability of the sequence.
b. Partitioning of the subset of pre-processed EOS sequences: For each time scale *p*, partition the original series into ⌊P/p⌋ non-overlapping subsets.
c. Subset statistical calculation: For each subset $Y_{k}=\left\{y_{\left(k-1\right)p+1},\ldots ,y_{kp}\right\}\;\left(k=1,2,\ldots ,n\right)$ perform the following calculations:
1. Calculate the mean (representing the average level of the EOS at this timescale):

(Equation 11) $$\mu_{k}=\frac{1}{p}\sum_{m=1}^{p}y_{\left(k-1\right)p+m}$$

2. Calculate the cumulative deviation (reflecting the cumulative trend of the sequence’s deviation from the mean):

(Equation 12) $$Z_{k,m}=\sum_{t=1}^{m}\left(y_{\left(k-1\right)p+t}-\mu_{k}\right)\left(m=1,2,\ldots ,p\right)$$

3. Calculate the range (characterizing the fluctuation range of EOS within the subset):

(Equation 13) $$R_{k}=\mathrm{Max}\left(Z_{k,m}\right)-\mathrm{Min}\left(Z_{k,m}\right)$$

4. Calculate the standard deviation (characterizing the dispersion of EOS within the subset):

(Equation 14) $$S_{k}=\sqrt{{\frac{1}{p}\sum_{m=1}^{p}\left(y_{\left(k-1\right)p+m}-\mu_{k}\right)}^{2}}$$

5. Calculate the rescaled standard deviation (to eliminate dimensions and enable comparison of fluctuations across different subsets):

(Equation 15) $${\left(R/S\right)}_{k}=R_{k}/S_{k}$$

d. Calculation of R/S mean values at each scale: For each time scale *p*, compute the mean value:

(Equation 16) $${\left(R/S\right)}_{p}=\frac{1}{n}\sum_{k=1}^{n}{\left(R/S\right)}_{k}$$

H fitting: Linear regression is performed with *log*((*R*/*S*)_*p*_) as the dependent variable and *log*(*p*) as the independent variable. The slope of this regression represents the H. To ensure computational accuracy, this fitting was implemented using MATLAB 2022b.

Subsequently, future EOS trends were derived based on the fitted H and the slope of EOS variation, with detailed classifications shown in Table 2.

### Quantification and statistical analysis

This study first preprocessed MODIS remote sensing data using MRT (https://lpdaac.usgs.gov/tools), then conducted statistical analyses employing Theil-Sen trend analysis, Mann-Kendall significance testing, partial correlation analysis, Geodetector (http://www.geodetector.cn/) and H. All analyses and spatial data processing were performed using ArcGIS 10.8.2 (https://www.esri.com/en-us/arcgis/) and MATLAB 2022b (https://www.mathworks.com/products/matlab.html), with *P* < 0.05 as the significance threshold. Theil-Sen trend analysis and Mann-Kendall test were employed to identify interannual trends and significance in vegetation phenology and hydrothermal factors within the QTP from 2001 to 2020. n = 20 represents the 20-year time series data from 2001 to 2020. Significance thresholds are referenced in Table 1, with results presented in Figures 2 and 3 and Table 3. Partial correlation analysis was employed to investigate the net correlation between vegetation phenology and hydrothermal factors, with results presented in Figure 5 and Table 4. Geodetector analysis assessed the explanatory power of single and interactive drivers of vegetation phenology, as shown in Figure 4. H was used to evaluate future trend characteristics of EOS in the study area, with classification reference in Table 2 and analysis results in Figure 6. The central tendency of each vegetation phenology and hydrothermal factor indicator in the study was represented by the arithmetic mean, while dispersion was quantified by SD. Trend analysis accuracy was assessed using 95% confidence intervals. All remote sensing data underwent outlier testing and missing value imputation to satisfy statistical analysis assumptions. Sample size was determined based on the availability and completeness of long-term remote sensing data in the study area, with no additional inclusion or exclusion criteria applied.

### Additional resources

All analyses in this study were conducted using publicly available remote sensing data and standard statistical software, without generating additional shareable datasets, analysis codes, tools, or supplementary resources. The open-source data sources utilized in the research are detailed in the methodology section, and the analytical procedures can be replicated using the quantitative and statistical methods described in the paper.
